# Eating behavior during pregnancy mediates the association between depression and diet quality--a new strategy for intervention in pregnancy

**DOI:** 10.3389/fpubh.2024.1339149

**Published:** 2024-02-08

**Authors:** Xingyi Jin, Jian Zhu, Niannian Wang, Lingzhen Sun, Junhui Yu, Shaokang Wang, Guiju Sun

**Affiliations:** ^1^Key Laboratory of Environmental Medicine and Engineering of Ministry of Education, Department of Nutrition and Food Hygiene, School of Public Health, Southeast University, Nanjing, China; ^2^Danyang Maternal and Child Health Hospital, Danyang, Zhenjiang, China; ^3^Department of Public Health, School of Medicine, Xizang Minzu University, Xianyang, China

**Keywords:** pregnancy, nutrient, depression, eating behavior, diet, mediation analysis

## Abstract

**Background:**

Depression can result in changes in eating behavior and decrease the quality of eating. It has been shown that maternal depression during pregnancy can result in malnutrition, which can have adverse effects on the pregnancy and the offspring. There is currently no clear association between depression and diet.

**Methods:**

Five hundred and forty-nine pregnant women recruited from Danyang Maternal and Child Health Hospital in Jiangsu Province participated in this study and were administered the Intuitive Eating Scale-2 (IES-2), Edinburgh Post-natal Depression Scale (EPDS), Pregnancy Stress Scale (PPS), Self-rating Anxiety Scale (SAS), and Dietary Guidelines Adherence Index for Pregnant Women during Pregnancy (CDGCI-PW). The nutritional software collected dietary records for three consecutive days in mid-pregnancy to calculate dietary intake and nutrients that support energy production. The mediation analyses were conducted using SPSS 24.0 macro PROCESS.

**Results:**

The relationship between depressive symptoms during pregnancy and diet quality was moderated primarily by two aspects of eating behavior, “Reliance on Hunger and Satiety Cues” (RHS) and “Body-Food Choice Congruence” (BFC). Depressive symptoms (EPDS scores) showed a negative correlation with RHS, BFC, and RHS, and BFC showed a positive correlation with diet quality, yielding a significant specific indirect effect. The multiple mediation model explained 14.7% of the variance in the diet quality.

**Conclusion:**

This study highlights the important role of eating behaviors during pregnancy in the relationship between depressive symptoms (EPDS scores) and diet quality, and provides preliminary evidence for feasible ways pregnant women with depressive symptoms can improve diet quality, promote maternal and child health, and reduce depression.

## 1 Introduction

During pregnancy, depression is one of the most common mental disorders (1–3). It can potentially impact the mother’s and unborn child’s health and well-being. The diet of mothers has been negatively impacted by depression according to several studies (4, 5). A recent cohort study of Chinese pregnant women found an inverse association between prenatal depression and mothers’ dietary patterns (6). Furthermore, poor diet quality is often detrimental to pregnant women. Pregnant women who consume low-quality diets are more likely to suffer from metabolic disorders and experience poor pregnancy outcomes (7–9). Moreover, it affects the development of their offspring (10, 11).

An irrational diet, obesity, and malnutrition are associated with gestational diabetes among Chinese mothers (12, 13). Research has demonstrated that pregnant women who consume higher-quality diets have offspring who possess better visual–spatial skills, intelligence, executive function, and behavior regulation abilities (14, 15). The importance of improving the diet of pregnant women is therefore paramount.

The importance of nutritional interventions during pregnancy has been demonstrated in numerous studies. In previous studies, the majority of nutrition interventions were dietitian-prepared meals, regular dietetic follow-up, and nutrient supplements. There has been little research on the motivation and changes in eating behavior of pregnant women themselves. Monitoring and developing healthy eating behaviors are equally important as a strategy for nutritional intervention during pregnancy. The Intuitive Eating Measurement Scale was developed by Tylka et al. to measure eating behavior and motivation. There are four different aspects of intuitive eating covered by the IES-2. “Unconditional Permission to Eat (UPE)” describes the behavior of eating whatever food is desired at the time. There is no classification of foods into allowed and forbidden items. UPE can be considered an anti-diet attitude. The “Eating for Physical Rather Than Emotional Reasons (EPR)” score assesses the ability to eat when physically hungry rather than relying on food to cope with negative emotions. According to EPR, eating styles are not influenced by emotional states. “Reliance on Hunger and Satiety Cues” (RHS) can be defined as the ability to regulate food intake by paying attention to hunger and satiety cues. RHS is characterized by the perception of physiological states. “Body-Food Choice Congruence” (BFC) refers to the selection of a healthy and tasty diet that is compatible with an individual’s bodily needs. The concept of gentle nutrition aims to honor the health and function of the body by extending intuitive eating (16). This study suggests that the IES scale should be understood as four related, but distinct dietary behaviors in a population of pregnant women rather than as a single score (17). Several studies have demonstrated that higher depressive states are associated with lower intuitive eating behaviors and that low eating behaviors result in lower dietary quality (17–19).

Currently, nutrition interventions are inconsistent, and many studies do not collect eating behavior changes. In other words, participants’ dietary perceptions or behaviors may change as soon as they enter a nutrition intervention study. Thus, the final outcome of the intervention may not be the result of just the intervention, but rather a combination of intuitive eating and positive eating accompanied by the intervention. The results of a recent study in 2023 indicate that simple changes in eating behavior alone can also influence nutritional quality without any intervention (20). An analysis of three European country pairs conducted in 2018 found an association between positive eating and both depressive symptoms and depressive disorders (21). Therefore, the purpose of this study was to investigate whether intuitive eating behaviors mediate the relationship between depressive states during pregnancy and diet quality in a population of pregnant women.

## 2 Materials and methods

### 2.1 Study design

The study was conducted at the Maternal and Child Health Hospital in Danyang City, Jiangsu Province, China. At the time of their first hospital visit following the diagnosis of pregnancy, all pregnant women in the region receive a Maternal and Child Health Card issued by the government. Women who were first-time cardholders were recruited and followed by nutritionists one-on-one during 20–25 weeks of gestation (when gestational diabetes screening was conducted). The sociodemographic information contained in the electronic records of the hospital is partial. The inclusion criteria for the study population were as follows: pregnant women diagnosed with a singleton pregnancy within the first 18 weeks of gestation; residing in Danyang and planning to give birth locally within the next year; voluntarily participating in the cohort study with informed consent; and capable of understanding and responding to the questionnaire accurately. The exclusion criteria were as follows: pregnant women under 18 years of age; pregnant women with previous diagnoses of syphilis, AIDS, anemia, cardiopulmonary disease, metabolic disorders, or psychological disorders; pregnant women with polycythemia vera. The study excluded 64 participants who were unable to complete the dietary questionnaire. A total of 549 pregnant women participated in this study between October 2022 and November 2023. They completed a baseline survey and a mid-pregnancy follow-up. The study was approved by the School of Public Health of Southeast University and Danyang Maternal and Child Health Hospital for ethical review.

### 2.2 Measures

#### 2.2.1 Intuitive eating

The IES-2 consists of 23 items that are rated on a 5-point Likert scale ranging from “strongly disagree” to “strongly agree” (16). A total of six items have been reverse-coded. There is an average score calculated for each of the four subscales and for the entire scale. The IES-2 was developed to assess four constructs thought to make up intuitive eating, including unconditional permission to eat (UPE; 6 items, e.g., “I tried to avoid certain foods high in fat, carbohydrates, or calories”), eating for physical rather than emotional reasons [EPR;8 items, e.g., “I usually stopped eating when I felt full (not overstuffed)”], reliance on hunger and satiety cues to determine when and how much to eat (RHS;6 items, e.g., “I could tell when I was slightly full”) and Body-Food Choice Congruence (BFC; 3 items, e.g., “I mostly eat foods that give my body energy and stamina.”). The Cronbach alphas for the Intuitive Eating-2 scale in the current sample were 0.78.

#### 2.2.2 Depression

Perinatal depression symptoms were evaluated according to the 10-item Edinburgh Postnatal Depression Scale (EPDS) (22). This is a self-reported scale originally developed for postpartum women and has since been validated for use with pregnant women to identify the risk for perinatal depression. The participants were asked to select the response that best reflected their current situation over the past 7 days. Each item was scored on a 4-point scale ranging from 0 to 3 (0 = most of the time/very often to 3 = never/not at all). Negatively stated items were reversed-scored so that higher scores indicated a higher level of perinatal depression. A total score above 13 indicates depression levels consistent with depressive disorders. In this analysis, we used the total EPDS score as a continuous measure of perinatal depression status within the last week. The depressive group was defined as those with EPDS scores greater than 13, and the healthy group was defined as those with EPDS scores below 13. The Cronbach alphas for the depression subscales of the EPDS in the current sample were 0.82.

#### 2.2.3 Anxiety

The widely used Zung’s Self-rating Anxiety Scale (SAS) was used to assess anxiety symptoms (23). Anxiety symptoms are measured by 20 self-reported items on the SAS. Some of the items were worded symptomatically positive rated on a 4–1 scale (a little of the time, some of the time, a good part of the time, and most of the time), and others symptomatically negative rated on a 1–4 scale. A standardized scoring algorithm defines anxiety symptoms, with a total score range of 20–80. A score of >50 on the SAS indicates anxiety disorder risk. In this analysis, we used the total SAS score as a continuous measure of perinatal anxiety status within the last week. The Cronbach alphas for the anxiety subscales of the SAS in the current sample were 0.85.

#### 2.2.4 Pregnancy stress

The Pregnancy Stress Scale (PPS), developed by Chan Changhui and some other researchers in the 1990s, was used to analyze the level and source of stress (24). There are 30 items in the PPS scale, which are categorized as follows: factor 1, stress induced by identification with parental roles, including 15 items; factor 2, stress induced by ensuring the health and safety of mother and child, including 8 items; factor 3, stress induced by physical activity and changes in appearance, including four items; factor 4, the remaining 3 items are other factors that have not been classified. There were four options for each item, including “not at all,” “a little,” “often,” and “always.” Scores were 0, 1, 2, and 3, and the total score was the sum of the scores for all entries, the higher the psychological stress, the higher the score. In this analysis, we used the total PPS score as a measure of perinatal stress status within the last week. The Cronbach alphas for the pregnancy stress subscales of the PPS in the current sample were 0.95.

#### 2.2.5 Pregnancy dietary quality

The Dietary Guidelines Adherence Index for Pregnant Women during Pregnancy (CDGCI-PW) is a clinical evaluation tool to establish and assess the dietary quality of pregnant women based on the Dietary Guidelines for Chinese Residents (2016). The mid and later-pregnancy dietary scale consists of 13 items, including 5 core questions and 8 general questions. According to the Chinese Dietary Guidelines for mid and late-pregnancy, the questionnaire includes all recommended food groups and intakes. Each question is scored according to the proportion of food in the dietary tower. The details of this scale can be found in other studies (25). In general, the higher the index scores, the higher the proportion of pregnant women who consume all types of food within the recommended range and the lower the proportion who consume less than the recommended range. Based on the total score of the scale, it was possible to understand the difference between the dietary intake of pregnant women and the national dietary recommendations, as well as evaluate the overall quality of a pregnant woman’s diet at mid and later pregnancy.

#### 2.2.6 Dietary intake assessment

An individual dietary assessment will be provided by a dietitian during each woman’s mid-pregnancy. During mid-pregnancy, pregnant women are required to bring their dietary records for three consecutive days to the hospital for their routine checkup. Recording researchers conducted a three-day, 72-h dietary review survey using the DaYingJia Intelligent Dietary Management System software, which determined the average daily dietary intake type, dietary intake, the intake of the three major energy-supporting nutrients (carbohydrates, proteins, and fats), and the percentage of energy supplied by those nutrients.

### 2.3 Statistical analysis

**Figure 1:**
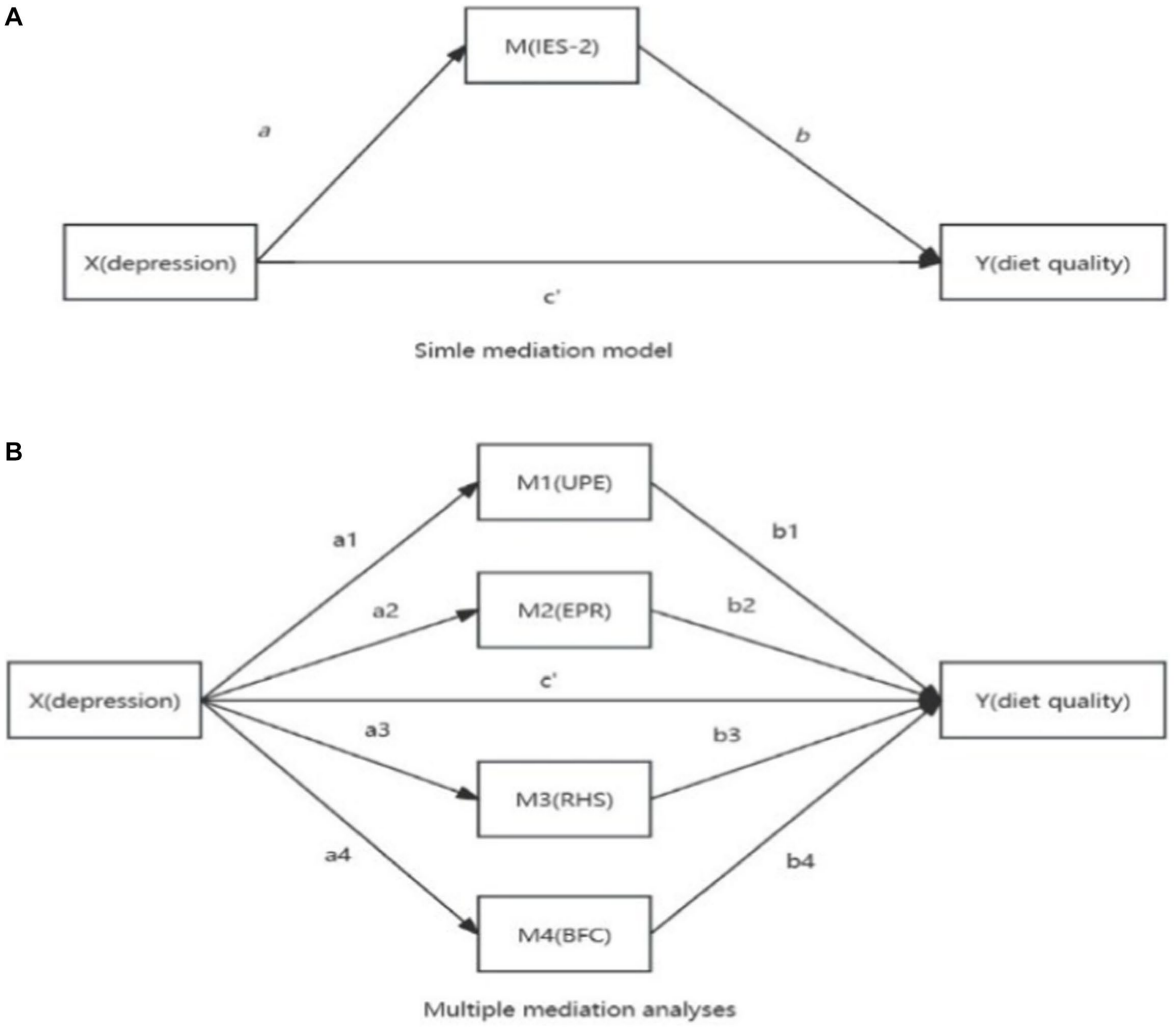
Analysis of two models (**A** and **B**).

Data were analyzed using Hayes’s (2013) PROCESS macro model 4 for SPSS 24.0 (26). Correlations were assessed by Pearson correlation coefficients between model variables (27) (27). A Bonferroni correction was conducted. There was no evidence of multicollinearity between the facets of intuitive eating (tolerance: 0.715–0.889, variance inflation factor: 1.124–1.398). Due to significant correlations between study variables and week, anxiety, and stress, these variables were included in the models as covariates of both the mediator and the outcome (26). Path analyses were conducted using ordinary least squares (OLS) regression analysis. Due to the IES-2 factor structure with four first-order latent factors and a second-order intuitive eating factor (16, 28), both a simple and a multiple mediation analysis were carried out. A multiple-mediator model has several advantages. It can provide a more accurate assessment of a mediation effect (29) and it allows the paths of each mediator to be examined with regard to the outcome while all other mediators are controlled (26). We used 10,000 bootstrap samples (percentile and 95% CI). Bootstrapping is a resampling method in which samples are repeatedly taken from the data set (with replacement) to estimate the sampling distribution of the indirect effect, and with that, to calculate the confidence interval for the indirect effect (26, 30). In contrast to normal theory approaches such as the Sobel test, bootstrapping does not make assumptions about the sampling distribution of the indirect effect. Therefore, a bootstrap confidence interval yields more accurate inferences and has more power (26, 31). If the 95% percentile bootstrap confidence interval for the indirect effect does not include zero, it is considered significant at the *p* < 0.05 level. Following Hayes (2013), unstandardized regression coefficients are reported. Specific analytical models can be found in Figures 1A,B.

## 3 Results

### 3.1 Baseline characteristics of study participants

In this study, a total of 549 individuals were included, of whom 242 had data on early pregnancy, mid-pregnancy, and late pregnancy, and 307 had data on mid-pregnancy only. Table 1 indicates that the mean age of the study participants was 30 years (SD = 4.81), the mean BMI level at mid-pregnancy was 24.60 kg/m^2^, and the mean gestation week at mid-pregnancy was 24.19 (SD = 2.2). In the study, 88.8% of participants completed secondary school or technical college, 59.1% had a household income ranging from 3,000 to 7,000 CNY per month, 63.9% worked, 90% had never smoked, 59.5% had never exercised, 95.6% took folic acid, 43.3% did not take any other nutritional supplements other than folic acid, 42.5% did not experience constipation, 62.1% slept regularly, and 25.8% considered themselves to have good sleeping quality. Furthermore, the mean scores for depressive symptoms (EPDS scores) (SAS score), anxiety (EPDS score), stress (PPS score), and diet quality (CDGCI-PW score) during mid-pregnancy were 5.1, 23.73, 23.03, and 3.46 (SD = 4.53, 6.98, 8.40, and 0.52 respectively).

**Table 1:** Baseline characteristics of the study sample of pregnant women (*n* = 549).

	Total	EPDS score ^c^ < 13 (*n* = 515)	EPDS score ^c^ ≥ 13 (*n* = 30)	*p*-value
	Mean [SD] or % (*n*)	Mean [SD] or % (*n*)	mean [SD] or % (*n*)	
**Age (years)**	30 [4.81]	31 [4.72]	29 [4.93]	0.451
**Height (m)**	160 [14.28]	159.15 [14.65]	160.36 [4.19]	0.652
**Weight before pregnancy (kg)**	59.49 [12.83]	59.58 [12.9]	55.25 [7.13]	0.112
**Weight in mid-pregnancy (kg)**	64.46 [10.97]	64.65 [10.88]	61.41 [13.08]	0.193
**Gestation week in early pregnancy (w)** ^**b**^	11.35 [2.08]	/	/	/
**Gestation week in mid-pregnancy (w)**	24.19 [2.2]	24.12 [1.94]	25.4 [1.99]	**<0.001**
**Education (%)**				0.677
Vocational high school and below	35 (6.4%)	34 (6.6%)	1 (3.3%)	
College and secondary school	483 (88.8%)	455 (88.3%)	28 (93.3%)	
Undergraduate and above	26 (4.8%)	25 (4.8%)	1 (3.3%)	
**Monthly disposable household income (%)**				0.741
More than 10,000 CNY	87 (15.9%)	84 (16.3%)	3 (10%)	
7,000–10,000 CNY	102 (18.7%)	93 (18.1%)	9 (30%)	
3,000–7,000 CNY	325 (59.1%)	310 (60.1%)	15 (50%)	
Less than 3,000 CNY	29 (5.3%)	26 (86.6%)	3 (10%)	
**Work (%)**				0.960
No	198 (35.8%)	186 (36.11%)	12 (40%)	
Yes	351 (63.9%)	333 (64.6%)	18 (60%)	
**Smoking (%)**				0.061
No, and no second-hand smoke	497 (90.5%)	468 (90.8%)	29 (96.6%)	
No, and have second-hand smoke	46 (9%)	46 (8.9%)	0 (0.0%)	
Used to smoke, but now quit	3 (0.5%)	2 (0.38%)	1 (3.33%)	
**Physical activity (%)**				0.461
No	327 (59.56%)	312 (60.5%)	15 (50%)	
Exercise all the time before pregnancy	82 (14.93%)	76 (14.75%)	6 (20%)	
Start exercising in early pregnancy	33 (9.45%)	26 (5.0%)	5 (16.6%)	
Start exercising in mid-pregnancy	107 (19.48%)	103 (20%)	4 (13.3%)	
**Body mass index before pregnancy (kg/m** ^**2**^ **)**	24.48 [9.74]	24.54 [9.83]	21.78 [2.64]	0.127
**Body mass index in mid-pregnancy (kg/m** ^**2**^ **)**	24.60 [4.83]	25.29 [4.81]	23.84 [5.05]	0.577
**Folic acid intake status (%)**				0.553
No	24 (4.4%)	22 (4.3%)	2 (3.3%)	
Yes	525 (95.6%)	497 (96.5%)	28 (93.3%)	
**Other nutrient intake (%)** ^**a**^				0.730
No	238 (43%)	226 (43.8%)	12 (40%)	
Yes	311 (57%)	293 (56.8%)	18 (60%)	
**Constipation (%)**				0.590
No	232 (42.5%)	220 (42.7%)	12 (40%)	
1–3 times per week	266 (48.2%)	252 (48.9%)	14 (46.6%)	
4–6 times per week	35 (6.4%)	32 (6.2%)	3 (10%)	
Every time	16 (2.9%)	15 (2.9%)	1 (3.3%)	
**Sleeping rhythm (%)**				0.060
Irregularly, with night shifts	16 (2.9%)	14 (2.7%)	2 (6.7%)	
Irregularly, sleeping at home at will	191 (35%)	172 (33.3%)	19 (63.3%)	
Regularly	342 (62.1%)	333 (64.0%)	9 (30%)	
**Depressive score in early pregnancy, EPDS score** ^**b**^	5.00 [5.15]	/	/	
**Depressive symptoms in early pregnancy, EPDS score** ^**b**^	17 (6.9%)	/	/	
**Depressive score in mid-pregnancy, EPDS score**	5.10 [4.53]	4.46 [3.73]	16.07 [2.62]	**<0.001**
**Depressive symptoms in mid-pregnancy, EPDS score**	30 (5.5%)	/	/	
**Anxiety score in mid-pregnancy, SAS score**	23.73 [6.98]	23.35 [6.78]	30.20 [7.36]	**<0.001**
**Anxiety symptoms in mid-pregnancy, SAS score**	1 (0.2%)	0	1	/
**Stress symptoms in mid-pregnancy, PPS score**	23.03 [8.45]	22.48 [7.9]	30.20 [7.73]	**<0.001**
**Diet quality in early pregnancy, CDGCI-PW score** ^**b**^	46.90 [17.65]	/	/	
**Diet quality in mid-pregnancy, CDGCI-PW score**	46.81 [14.24]	42.86 [14.68]	37.47 [12.92]	**0.044**
**Diet quality in late pregnancy, CDGCI-PW score** ^**b**^	67.86 [9.77]	/	/	
**Intuitive Eating Scale-2 score in mid-pregnancy**	3.46 [0.52]	3.48 [0.49]	3.33 [0.57]	0.068
UPE	3.24 [0.54]	3.25 [0.53]	3.17 [0.64]	0.047
EPR	3.11 [0.43]	3.13 [0.38]	3.10 [0.43]	0.074
RHS	3.71 [0.58]	3.73 [0.52]	3.43 [0.57]	**0.005**
BFC	3.80 [0.57]	3.81 [0.56]	3.60 [0.65]	**0.046**

SD, standard deviation; EPDS, Edinburgh Post-natal Depression Scale; PPS, Pregnancy Stress Scale; SAS, Self-rating Anxiety Scale; CDGCI-P, dietary guidelines adherence index for pregnant women during pregnancy; IES2, Intuitive Eating Scale-2; EPR, eating for physical rather than emotional reasons; UPE, unconditional permission to eat; RHS, Reliance on hunger and satiety cues; BFC, body-food choice congruence.

^a^Did not take any other nutritional supplements besides folic acid.

^b^*n* = 246 due to missing values.

^c^Based on mid-pregnancy EPDS score.

Bold indicates *p* < 0.05.

### 3.2 Depressive symptoms and diet

Further research was conducted on 246 pregnant women who completed a recorded dietary questionnaire mid-pregnancy for three days (72 h) in Table 2. The results of the study showed that 17 pregnant women experienced significant depressive symptoms (EPDS score ≥ 13) during their pregnancy. A statistically significant difference was not found between the two groups of pregnant women at baseline (age, gestational week, pre-pregnancy body mass index, current body mass index, literacy level, and economic level), see Table 1. Compared to pregnant women without mid-pregnancy depressive symptoms, pregnant women with mid-pregnancy depressive symptoms consumed a more varied diet (*p* = 0.02, higher fruit intake (*p* = 0.006), more livestock and meat (*p* = 0.043), and fewer soybeans and nuts (*p* = 0.001)). There was no significant difference between the two groups in terms of the proportion of energy supplied by carbohydrates, proteins, and fats. However, both total carbohydrate and protein intake were significantly higher in the depressed group than in the health group (*p* = 0.005,0.016). Depressive symptoms (EPDS scores) scored lower on the “Reliance on Hunger and Satiety Cues” scale (*p* = 0.032). The quality of diet was worse in both mid and late-pregnancy than in the health group (*p* = 0.05, 0.006).

**Table 2:** Associations between depressive symptoms and eating status, intuitive eating behaviors.

	Health group (*n* = 229)	Depression group^a^ (*n* = 17)	*P-value*
**Number of species**			
Daily Ingredients	14.31 (5.91)	21 (9.35)	**0.020**
Grains	2.82 (1.76)	3.8 (1.92)	0.233
Vegetables and fruits	4.3 (1.97)	6 (2.34)	0.067
Meat, fish and seafood	2.63 (1.22)	2.8 (1.30)	0.790
Dairy, legumes, and nuts	1.48 (1.03)	1.60 (1.51)	0.801
**Food intake (g)**			
Grains	301.99 (183.37)	384.26 (200.18)	0.335
Potatoes	63.18 (54.83)	103.5 (65.32)	0.243
Vegetables and mushrooms	200.46 (153.54)	194.88 (277.71)	0.940
Fruits	214.11 (115.86)	389 (169.80)	**0.006**
Livestock and poultry meat	120.39 (113.68)	257.76 (95.34)	**0.043**
Egg	82.97 (41.40)	76.02 (32.83)	0.716
Soybeans and nuts	89 (1.5)	7.75 (8.9)	**0.000**
Aquatic products	86.78 (68.31)	167.4 (215.8)	0.183
Milk and dairy products	233.77 (89.31)	313.87 (134.11)	0.107
Fast food	81.16 (78.97)	92.2 (70.42)	0.848
**Intake of energy-supporting nutrients**			
Carbohydrates as a percentage of total energy	54.07 (8.86)	58.23 (11.08)	0.317
Protein as a percentage of total energy	17.90 (3.20)	18.40 (4.71)	0.748
Fat as a percentage of total energy	28.01 (7.98)	23.36 (10.37)	0.216
Carbohydrate intake	218.55 (68.74)	313.62 (113.80)	**0.005**
Protein intake	69.93 (21.06)	95.46 (42.20)	**0.016**
Fat intake	50.28 (22.89)	52.33 (28.5)	0.848
**IES-2 score in mid-pregnancy**	16.86 (2.19)	16.64 (2.06)	0.610
UPE	20.12 (1.87)	20.14 (2.03)	0.977
EPR	24.18 (3.63)	25.57 (2.50)	0.321
RHS	11.52 (1.63)	10.14 (1.57)	**0.032**
BFC	11.63 (1.64)	10.71 (2.14)	0.166
**Quality of diet (CDGCI-PW score)**			
Early Pregnancy	48.05 (19.94)	47.50 (20.31)	0.947
Mid-Pregnancy	52.19 (14.70)	41.43 (11.97)	**0.050**
Late Pregnancy	69.67 (9.85)	57.00 (10.65)	**0.006**

SD, standard deviation; CDGCI-PW, dietary guidelines adherence index for pregnant women during pregnancy; IES-2, Intuitive Eating Scale-2; EPR, eating for physical rather than emotional reasons; UPE, unconditional permission to eat; RHS, Reliance on hunger and satiety cues; BFC, body-food choice congruence.

^a^Depression = EPDS score > 13.

Bold indicates *p* < 0.05.

### 3.3 Intuitive eating behavior and diet quality

Table 3 shows the association between intuitive eating behaviors during mid-pregnancy and diet quality in mid and late-pregnancy. According to the crude model, mid-pregnancy intuitive eating behavior scores, UPE scores, EPR scores, and RHS scores were significantly related to late-pregnancy diet quality (*p* = 0.029, 0.015,0.032, 0.018). There was a significant association between the mid-pregnancy RHS score and the BFC score (*p* = 0.050, 0.031) after adjustment for confounders. There was a significant association between the total intuitive eating score, the UPE score, and the RHS score in late pregnancy (*p* = 0.049, 0.028, and 0.006).

**Table 3:** Association of intuitive eating behaviors in mid-pregnancy with diet quality in mid and late-pregnancy.

	Crude model^a^			Adjusted model^b^				
	Beta	*t*	*p*	Beta	*t*	*p*	95%CI	
**Mid-pregnancy intuitive eating behaviors and mid-pregnancy diet quality**								
IES-2	−0.302	−0.625	0.533	−0.618	−1.111	0.269	−1.918	0.541
UPE	−0.002	−0.012	0.991	0.167	0.769	0.443	−1.931	4.377
EPR	0.243	0.811	0.419	0.322	0.941	0.349	−1.303	3.655
RHS	0.306	1.938	0.055	0.315	1.864	**0.050**	0.168	5.380
BFC	0.244	1.437	0.153	0.405	2.187	**0.031**	0.312	6.415
**Mid-pregnancy intuitive eating behaviors and late-pregnancy diet quality**								
IES-2	−1.190	−2.218	**0.029**	−1.243	−1.935	**0.049**	−2.007	−0.027
UPE	0.504	2.462	**0.015**	0.546	2.238	**0.028**	0.330	5.576
EPR	0.723	2.175	**0.032**	0.686	1.738	0.086	−0.260	3.868
RHS	0.448	2.403	**0.018**	0.460	2.777	**0.006**	0.801	4.788
BFC	0.214	1.164	0.247	0.272	1.291	0.200	−0.875	4.121

IES2, Intuitive Eating Scale-2; EPR, eating for physical rather than emotional reasons; UPE, unconditional permission to eat; RHS: Reliance on hunger and satiety cues; BFC, body-food choice congruence.

^a^Crude model did not adjust for any confounding factors. ^b^Adjusted model adjusted for age, week, education, prenatal BMI, mid-pregnancy BMI, folic acid intake status, constipation, sleeping rhythm, sleeping quality, smoke, anxiety, and stress.

Bold indicates *p* < 0.05.

### 3.4 Association of depression, intuitive eating, and diet quality

We further mediated the analysis with 549 pregnant women who provided mid-pregnancy information to assess the associations between depressive symptoms (EPDS scores), intuitive eating behaviors, and diet quality. The following Table 4 presents a summary of descriptive statistics and correlational data. There was a significant negative correlation between depressive symptoms and stress and the RHS and BFC. UPE and anxiety were significantly correlated. There was a significant positive correlation between diet quality and the IES-2 total score, RHS, and BFC, and a significant negative correlation between diet quality and depressive symptoms and anxiety. A significant correlation was found between depressive symptoms and gestation week among the sociodemographic variables. There was no association between BMI during pregnancy and variables (IES-2, EPR, UPE, RHS, BFC, depressive symptoms (EPDS scores), stress, anxiety, diet quality, GWG, and week).

**Table 4:** The intercorrelations between model variables.

	1	2	3	4	5	6	7	8	9	10	11	12
1. IES-2	1											
2. EPR	0.827^**^	1										
3. UPE	0.508^**^	0.330^**^	1									
4. RHS	0.542^**^	0.147^**^	0.012	1								
5. BFC	0.632^**^	0.270^**^	0.067	0.495^**^	1							
6. Depression	−0.046	0.075	0.046	−0.208^**^	−0.138^**^	1						
7. Stress	−0.037	0.057	0.041	−0.171^**^	−0.104^*^	0.490^**^	1					
8. Anxiety	0.045	0.086^*^	−0.015	−0.041	0.031	0.321^**^	0.509^**^	1				
9. Diet quality	0.223^**^	0.078	0.032	0.266^**^	0.287^**^	−0.145^**^	−0.080	0.139^**^	1			
10.GWG	0.067	0.078	0.006	0.058	0.001	0.077	0.139	0.090	−0.046	1		
11. Week	−0.037	−0.002	−0.004	−0.070	−0.046	0.109^*^	0.076	0.029	−0.042	0.064	1	
12. BMI (mid-pregnancy)	−0.047	−0.024	0.007	−0.079	−0.036	−0.049	−0.041	−0.042	−0.014	0.120	−0.026	1

IES2, Intuitive Eating Scale-2; EPR, eating for physical rather than emotional reasons; UPE, unconditional permission to eat; RHS, reliance on hunger and satiety cues; BFC, body-food choice congruence; GWG, gestational weight gain; BMI, body mass index.

∗∗Correlation is significant at the 0.01 level (2-tailed).

∗Correlation is significant at the 0.05 level (2-tailed).

#### 3.4.1 Simple mediation analyses

The results of simple mediation analysis are shown in Table 5. A significant positive association was found between IES-2 and diet quality and a significant negative relationship was found between depressive symptoms and diet quality when anxiety, stress, and gestational week were taken into account. There was no association between depressive symptoms (EPDS scores) and IES-2, however. As the percentile bootstrap confidence interval contained zero, the indirect effect was not statistically significant.

**Table 5:** Results for the pathways and model summary information in the simple.

		IES2 (M)				DQ (Y)		
		coeff	*t*	*p*		coeff	*t*	*p*
Depression (X)	a	−0.053	−0.847	0.397	*c*’	−0.482	−3.269	0.001
IES (M)		/	/	/	*b*	0.496	4.913	<0.001
Cov (week)		−0.089	−0.714	0.475		−0.116	−0.394	0.693
Cov (stress)		−0.040	−1.083	0.279		−0.199	−2.280	0.023
Cov (anxiety)		0.073	1.778	0.075		0.487	5.034	<0.001
								
		*R* ^2^	0.009			*R* ^2^	0.110	
		*F*	1.251			*F*	13.374	

IES2, Intuitive Eating Scale-2; DQ, diet quality.

#### 3.4.2 Multiple mediation analyses

Based on previous research, the IES-2 scale is not likely to lead to uniform behavior in pregnant women (the total score is meaningless), but instead should be divided into four subscales. This has also been demonstrated by other special groups. As a result, we divided the IES-2 scale into four subscales and conducted multiple mediation analyses.

The multiple mediation analysis, after controlling for gestational week, anxiety, and stress, indicated a significant total indirect effect (Table 6). In the relationship between depressive symptoms during pregnancy and diet quality, RHS and BFC were the primary moderators. Depressive symptoms showed a negative correlation with RHS, BFC, and RHS, and BFC showed a positive correlation with diet quality, yielding a significant specific indirect effect. The confidence interval for the contrast between the specific indirect effects of EPR and UPE included zero, indicating that the specific indirect effects of EPR and UPE did not statistically differ from each other. Meanwhile, depressive symptoms showed a negative correlation with diet quality (Table 7). Therefore, it is an indirect mediation model and the multiple mediation model explained 14.7% of the variance in the diet quality.

**Table 6:** Indirect effects of depression on diet quality through the facets of intuitive eating.

			95% Percentile confidence interval	
Mediator	Parameter estimate	SE	Lower	Upper
Total	−0.144	0.053	−0.255	−0.045
UPE	−0.004	0.010	−0.011	0.032
EPR	−0.001	0.010	−0.024	0.020
RHS	−0.076	0.035	−0.157	−0.020
BFC	−0.070	0.035	−0.151	−0.010

EPR, eating for physical rather than emotional reasons; UPE, unconditional permission to eat; RHS, reliance on hunger and satiety cues; BFC, body-food choice congruence.

**Table 7:** Results for the pathways and model summary information in the multiple mediation model to explain diet quality.

	M1 (UPE)				M2 (EPR)				M3 (RHS)				M4 (BFC)				Y (DQ)			
Antecedent		coeff.	SE	*p*		coeff.	SE	*p*		coeff.	SE	*p*		coeff.	SE	*p*		coeff.	SE	*p*
X (depression)	a_1_	0.014	0.017	0.423	a_2_	0.043	0.038	0.257	a_3_	−0.0636	0.0184	**0.001**	a_4_	−0.047	0.018	**0.011**	*c*’	−0.509	0.150	**0.001**
M_1_ (UPE)		/	/	/		/	/	/		/	/	/		/	/	/	*b*_1_	0.288	0.370	0.437
M_2_ (EPR)		/	/	/		/	/	/		/	/	/		/	/	/	*b*_2_	−0.033	0.179	0.855
M_3_ (RHS)		/	/	/		/	/	/		/	/	/		/	/	/	*b*_3_	1.197	0.384	**0.002**
M_4_ (BFC)		/	/	/		/	/	/		/	/	/		/	/	/	*b*_4_	1.504	0.391	**0.001**
Cov (week)		−0.008	0.035	0.814		−0.017	0.076	0.8205		−0.0393	0.0369	0.288		−0.025	0.037	−0.663		−0.075	0.291	0.796
Cov (stress)		0.009	0.011	0.371		−0.002	0.023	0.9075		−0.0257	0.0109	0.019		−0.022	0.011	0.052		−0.159	0.086	0.066
Cov (anxiety)		−0.012	0.011	0.295		0.035	0.025	0.1554		0.019	0.0121	0.115		0.031	0.012	0.012		0.459	0.095	<0.001
		R^2^	0.005			*R* ^2^	0.019			*R* ^2^	0.0559			*R* ^2^	0.033			*R* ^2^	0.148	
		F	0.631			*F*	1.357			*F*	8.0123			*F*	4.636			*F*	11.601	
		P	0.641			*P*	0.247			*P*	**<0.001**			*P*	**<0.001**			*P*	**<0.001**	

EPR, eating for physical rather than emotional reasons; UPE, unconditional permission to eat; RHS, reliance on hunger and satiety cues; BFC, body-food choice congruence.

Bold indicates *p* < 0.05.

## 4 Discussion

This study investigated whether eating behaviors during pregnancy are associated with depressive symptoms and diet quality in pregnant women for the first time. In this study, preliminary simple mediation analyses indicated there was no relationship between intuitive eating behaviors, depressive symptoms (EPDS scores), and diet quality. According to previous studies, Daundasekara et al. concluded that subscales should be used rather than the total scale score to describe pregnant women’s eating behaviors (17). Therefore, we conducted multiple analyses. The results indicated that the RHS and BFC mediated the relationship between depressive symptoms and diet quality and that depressive symptoms can directly influence diet quality. The relationship between depressive symptoms and diet quality was mediated by certain eating behaviors during pregnancy.

In the association between depressive symptoms and dietary intake, our study showed the opposite results to previous studies, which indicated that depressive symptoms were lower in those with high fruit and vegetable intake (32–34). But our research shows, the depressed group consumed more fruits and food groups than the health group but consumed less legumes. There was no difference between the depressed and health groups regarding the three macronutrient energy supply ratios, but carbohydrate and protein intake was greater in the depressed group. Furthermore, women who were classified as depressed in mid-pregnancy had poorer diet quality in the early, mid, and late stages of pregnancy compared to the health group. Even though the diet quality of the two groups was similar in the early stages of pregnancy, a significant difference was observed in the late stages. In addition, intuitive eating behaviors have an impact on the quality of the diet during the second and third trimesters of pregnancy. As a result, according to previous research, individuals with high levels of neuroticism are highly likely to engage in binge eating behaviors (35). Although eating disorder behaviors were not measured in our study. However, based on the diet of the depressed group, we found that carbohydrates and proteins were significantly higher, while the energy supply ratio of both was the same. Moreover, the depressed group consumes a greater variety of diets, but scores lower in terms of quality. Therefore, we presumed that the depressed group had a higher and unhealthy dietary intake and was more likely to have binge eating symptoms.

Although studies have shown that people with high fruit intake have less depression, most studies have not set a maximum fruit intake and have used people with low fruit intake as controls. In addition, some of the studies did not examine vegetables and fruits separately but combined them into one option. Though fruit is considered a “healthy food” in common sense, excessive consumption of fruit does not constitute a “healthy” diet. The consumption of high glycemic index fruits, in particular, can result in an increase in blood sugar levels or even the development of gestational diabetes in pregnant women. Women who are pregnant may pay more attention to “healthy” foods than those who are not pregnant. The general population may select ultra-processed sweets when overeating is prevalent, while pregnant women may prefer “healthy” sweets like fruit. A study of pregnant women showed that sweet consumption was more reflective of a tendency to ignore satiety (36). As a result, we do not believe that it is reasonable to combine vegetables and fruits on this point, as they differ in this regard. Those who suffer from depression and lose their appetite may also be more inclined to consume sugary foods rather than fewer vegetables (37, 38).

It has been shown in our research that negative emotions also influence eating behaviors and that all three kinds of emotions – depression, anxiety, and stress – influence each other. This is consistent with previous research (39). Although depression anxiety stress are all negative emotions, the three are not the same (40). In our study, both depressive symptoms and stress scores were correlated with RHS and BFC scores, but anxiety scores were only correlated with EPR scores. It has been demonstrated in previous studies that binge eating is more common in individuals suffering from depression or anxiety and less common in more stressed individuals (41, 42). This may be due to the fact that our study measured pregnancy stress. Among pregnant women, stress from pregnancy might be a contributing factor to the development of depression. Therefore, the consistency of the association was reflected in this study. In addition, our study did not observe the effects of gestational weight gain, gestational week, BMI, and eating behaviors, but there was an association between gestational week and depressive symptoms. The reason for this may be that the weight gain during pregnancy was not significant. Nevertheless, the gestational week is a reflection of the course of pregnancy. As time passes, depressive symptoms may become more pronounced as we reach the latter stages of pregnancy. The risk of prenatal depression increases as the pregnancy progresses and clinically significant depressive symptoms are common in the mid and late trimester (43). In order to further understand the importance of observing this part in the latter stages of pregnancy, it is necessary to differentiate between different trimesters of pregnancy.

According to our study, depressive symptoms (EPDS scores) is associated with fewer intuitive eating behaviors and poorer diet quality, as well as fewer intuitive eating behaviors associated with poorer diet quality. Depressive symptoms have been shown to influence eating behaviors such as emotional eating and eating disorders (44). Furthermore, they may not be able to follow their self-imposed hunger to eat or stop eating, which can also be reflected in the RHS scale. However, for mothers who are unable to reduce their intake of food and tend to reject unhealthy, high-sugar foods, conflicting eating behaviors may result. Depression during pregnancy and diet may be biologically related (45). In pregnancy, depression scores have been associated with reductions in dairy products, sweetened beverages, sugar, and packaged snacks, increased magnesium intake, increased non-core foods intake, and decreases in core food and fruit intake (4). Due to this, BFC was more evident during the same time period in studies of pregnant women participating in intuitive eating programs. Furthermore, our study showed that lower intuitive eating behaviors in mid-pregnancy predicted poorer diet quality in late pregnancy. Most current studies, however, are cross-sectional and cannot predict future diet quality because they are limited to contemporaneous data.

Previous studies have focused primarily on depression and diet quality without considering dietary behaviors within the population (46, 47). Our study demonstrated that, during mid-pregnancy, pregnant women’s RHS and BFC eating behaviors were associated with depressive symptoms (EPDS scores) and diet quality. The results of a recent study suggest that eating behaviors may mediate the relationship between satiety and dietary intake and that interventions targeting hunger and satiety may help prevent binge eating (48). As part of our study, the RHS was a subscale measuring whether pregnant participants adhered to hunger and satiety during mid- and late pregnancy, with the RHS demonstrating a significant association with diet quality during this period. During pregnancy, women undergo significant physical, physiological, and immunological changes, making them more susceptible to nutritional deficiencies (4). When women are not pregnant, they focus on maintaining a healthy body image and self-image, and they may be more concerned about their hunger to avoid nutritional deficiencies. As a result, pregnant women may be more inclined to follow their body hunger. Other similar studies have focused on the mediating role of positive eating behaviors (49, 50). A recent study found a similar relationship between intuitive eating and positive thinking, for example in choosing healthy foods (51). Therefore, our study provides some support for previous mediational modeling studies that suggest positive-thought eating mediates the association between negative emotions and eating.

In addition, nutritional psychiatry is emerging as a very promising field, and numerous studies have demonstrated that nutrition can improve mental health. As a result, reverse causality may exist (52, 53). In a study by Namely et al., people who had previously suffered from depression and sought treatment scored higher on eating patterns, and those unaware of their depression scored lower. Participants in our study were pregnant women who had never been screened for depressive symptoms (EPDS scores) and who had not received any psychological assistance before participation. Therefore, our study falls under the category of assessing the effect of depressive symptoms on eating behavior and dietary intake without knowing whether a person is depressed. In some studies, the results may not have been significant because some of the study populations knew they had depressive symptoms before being observed in their eating behaviors, so they may have altered their eating behaviors subjectively. This resulted in confounding and did not reflect depression’s impact on both. The effects of nutritional interventions may be bidirectional in the real world. In our opinion, this may not matter, since improved eating behaviors could reduce the negative effects of depressive symptoms during pregnancy while improving the quality of the diet during pregnancy and encouraging the development of healthy children. In addition to traditional nutritional interventions, we believe pregnant women should be educated about eating behaviors. These two strategies are essential for ensuring good nutritional status during pregnancy and promoting the health of the mother and child. The approach is also more economically advantageous from a socio-economic standpoint.

Our limitations are, first, that while we collected diet quality during pregnancy, we did not measure dietary behaviors in late pregnancy. As a result, even though we observed a correlation between dietary behaviors in mid-pregnancy and diet quality during late pregnancy, we were not able to find a correlation between dietary behaviors during late pregnancy and diet quality. As a result of the special characteristics of pregnancy, pregnant women’s dietary behaviors may change more rapidly than the general population during pregnancy. Consequently, we do not believe that mid-pregnancy dietary behaviors can replace late-pregnancy dietary behaviors. In addition, we did not measure emotional eating, binge eating behavior, or personality traits in pregnant women. The results of the study indicate that people with severe depressive symptoms are highly likely to experience binge eating disorder symptoms. Eating behaviors influenced by personality traits were not assessed in this study. We also failed to measure social family support for pregnant women, as this can also affect depression. Lastly, the limitation of this study is the lack of a clinical diagnosis of depression (54). However, the EPDS is a widely used screening tool for postpartum depression, having been validated against clinical interviews in the setting where the present study was carried out, and with good psychometric properties (54). In the future, we believe that it is possible to conduct prospective studies in the early, middle, and late phases of pregnancy, as well as during the postpartum period, which will allow us to observe the changes in dietary behaviors over time. Consequently, it is possible to obtain more convincing evidence to discuss the effects of depression during pregnancy on eating behavior and quality, as well as to investigate the bidirectional causal role that nutrition plays in depression. The effectiveness of traditional interventions can be compared to develop new strategies for pregnancy interventions in the future.

## 5 Conclusion

This study highlights the important role of eating behaviors during pregnancy in terms of the relationship between depression and diet quality and provides preliminary evidence for possible ways to achieve higher diet quality in pregnant women with depressive symptoms. In particular, for pregnant women with some depressive tendencies, promoting maternal and offspring health by encouraging intuitive eating behaviors as a nutritional intervention is a useful and economical strategy. Future studies of intuitive eating behaviors could be conducted in more pregnant women populations to gain more insight into the temporal and causal relationships between depression and diet quality.

## Data availability statement

The raw data supporting the conclusions of this article will be made available by the authors, without undue reservation.

## Ethics statement

The studies involving humans were approved by the School of Public Health of Southeast University and Danyang Maternal and Child Health Hospital for ethical review. The studies were conducted in accordance with the local legislation and institutional requirements. The participants provided their written informed consent to participate in this study.

## Author contributions

XJ: Data curation, Investigation, Methodology, Software. JZ: Writing – review & editing, Funding acquisition, Investigation, Software, Supervision. NW: Conceptualization, Investigation, Software. LS: Writing – review & editing, Conceptualization, Investigation, Software. JY: Writing – review & editing, Conceptualization, Investigation, Software. SW: Writing – review & editing, Funding acquisition, Project administration, Resources, Validation, Visualization. GS: Writing – review & editing, Project administration, Validation, Visualization.
